# The effects of endometrial thickness on outcomes of pregnancy following embryo transfer: A retrospective cohort

**DOI:** 10.5935/1518-0557.20240016

**Published:** 2024

**Authors:** Aghdas Safari, Foruheh Moazzezi, Mohammad Azizi

**Affiliations:** 1 Imam Reza Hospital, Department of Obstetrics and Gynecology, Faculty of Medicine, Aja University of Medical Sciences, Tehran, Iran; 2 Department of General Surgery, Shahid Modarres Hospital, Faculty of Medicine, Shahid Beheshti University of Medical Sciences, Tehran, Iran; 3 Be’sat Hospital, Faculty of Medicine, Aja University of Medical Sciences, Tehran, Iran

**Keywords:** assisted reproduction, embryo transfer, endometrial thickness, birth weight, preterm labor, pregnancy

## Abstract

**Objective:**

The safety of assisted reproductive technology can be assessed by examining birth weight as an outcome measure. The objective of this study was to evaluate the effect of endometrial thickness during embryo transfer on newborn birth weight and preterm labor.

**Methods:**

We conducted a retrospective cohort study at the infertility department of a teaching hospital affiliated with a university of medical sciences. Eligible women were ≥18 years old and conceived a singleton pregnancy with embryo transfer and an endometrial thickness of ≥7 mm. None of the patients had diabetes, blood hypertension, and polycystic ovarian syndrome. We assessed maternal and newborn characteristics and perinatal pregnancy outcomes.

**Results:**

In total, 100 eligible patients with a mean (SD) age of 32.8 (6.2) years were included. The mean endometrial thickness during embryo transfer was 9.1 (1.2) mm, and the mean birth weight was 3040.7 (565.3)g. There were no statistically significant associations between endometrial thickness and preterm labor (*p*=0.215) and between endometrial thickness and stillbirth or intra-uterine fetal death (*p*=0.880). However, after adjusting for confounding factors, the association of endometrial thickness with birth weight was statistically significant [b=124.6 (51.6), *p*=0.018].

**Conclusions:**

Within the range of ≥7mm, endometrial thickness during embryo transfer is a predictor of newborn weight; however, it is not related to the risk of preterm labor, stillbirth, or intra-uterine fetal death.

## INTRODUCTION

The use of assisted reproductive technology (ART) has increased substantially over time, with ART procedures now accounting for 1.5 to 5.9% of all births in high-income countries (da Silva *et al.*, 2020). However, some studies have suggested that ART singletons are at an increased risk of preterm birth, low birth weight (LBW), congenital anomalies, and perinatal mortality (Banica *et al*., 2021; Chang *et al*., 2020). Despite these risks, the number of pregnancies conceived through ART continues to grow (Guo *et al*., 2020). The effects of ART on newborn health remain an open question (da Silva *et al*., 2020), as early life events are believed to play a crucial role in modulating the risk of certain diseases during later periods (Mohammadi *et al*., 2023; Nobile *et al*., 2022). Meanwhile, ART has undergone considerable changes over the last decade as well (Chang *et al*., 2020; Kushnir *et al*., 2017; Reig & Seli, 2019). Consequently, research is still needed to investigate the association of ART with pregnancy outcomes (da Silva *et al*., 2020).

Studies have shown that endometrial thickness (EMT) is associated with the outcomes of pregnancies conceived through ART (Kasius *et al*., 2014). A thicker endometrium is believed to be beneficial for proper implantation and the ultimate success of ART procedures (Banker *et al*., 2021). However, there is no clear consensus on the ideal EMT for successful pregnancy outcomes, with varying opinions and recommendations among fertility specialists (Mahajan & Sharma, 2016). Many studies suggest that a thickness of 7 mm or more is associated with a high chance of pregnancy success (Liu *et al*., 2018; Wang *et al.*, 2018), while others have found that a thickness of 9 mm or more is ideal (Zhao *et al*., 2012). More data are required to enhance comprehension of the precise correlation between EMT and ART outcomes, thereby enabling the development of more effective fertility interventions (Kasius *et al*., 2014; Mahajan & Sharma, 2016). For the safety evaluation and standardization of the practice, the perinatal outcomes of ART still require careful evaluation (Beltran Anzola *et al*., 2019; Kushnir *et al*., 2017).

We conducted this study to evaluate the effect of EMT on the outcomes of ART. Our hypothesis was that EMT affects birth weight (BW) and the risk of preterm delivery. We incorporated a number of maternal characteristics into our statistical models to control their confounding effects on the relations between EMT and ART outcomes.

## METHODS

### Design and setting

This study was a retrospective cohort analysis of deliveries following ART between 2018 and 2022. All patients were treated for infertility at the Department of Infertility at a teaching hospital affiliated with a University of Medical Sciences. The department is well-equipped and the hospital is a large referral and subspecialty center.

### Ethics approval

The study protocol was conducted in accordance with the Declaration of Helsinki. Ethics approval was obtained from the Institutional Review Board (IRB) of the University of Medical Sciences. The study used information from participants’ medical records and did not involve any measurements on patients. No identifying information was extracted from patients’ records. All participants provided written informed consent for their treatment and received verbal and written explanations of the nature and purpose of the procedures.

### Eligibility

The study included women aged 18 years or older who had conceived a singleton pregnancy of 22 weeks or more or weighing 500g or more using ART. Based on the available information, the procedures were performed for patients with tubal factor infertility, polycystic ovary syndrome, light to moderate decreased semen quality in the male partner, or unexplained infertility. None of the included patients had ovarian cysts larger than 4 cm, hypogonadotropic hypogonadism, endometriosis stage 3-4, and liver, kidney, or thyroid disease. The procedures were not prescribed for women with severe diabetes, cardiovascular disease, or other comorbidities that interfered with the treatments. Women who developed gestational hypertension or diabetes, those who underwent previous in-vitro fertilization or intracytoplasmic sperm injection with their current partner, and users of donor oocytes or frozen oocytes were excluded as well. Patients with incomplete records were also excluded.

### Protocols and Procedures

#### ART

We used controlled ovary stimulation for ICSI and gonadotropin-releasing hormone agonists or antagonists to prevent premature ovulation. Follicle-stimulating hormone injections were administered until the follicular diameter of 18 mm was reached (Huang *et al*., 2022; Ribeiro & Sousa, 2023; Rodriguez-Wallberg *et al*., 2022). A 10000-unit human chorionic gonadotropin (hCG) was injected and after 36 hours the oocytes were retrieved using a transvaginal ultrasound probe to guide a specialized needle through the vaginal wall and into the ovaries and were fertilized with husband sperms. If endometrium was at least 7 mm, progesterone was administered for 3 or 5 days and then the embryo or blastocyst was transferred. For frozen-ET, retrieved oocytes were fertilized in the laboratory with ICSI and the embryo was cryopreserved using vitrification (Arian *et al*., 2023; Lee *et al*., 2022; Roelens & Blockeel, 2022). The endometrium was primed with estradiol until the EMT reached at least 7 mm, progesterone was administered for 3 days, and the frozen embryo was thawed and transferred into the uterus using a flexible catheter under ultrasound guidance. Pregnancy was investigated two weeks later using a serum beta-hCG test and sonography.

#### Endometrial thickness

The EMT (mm) was assessed by measuring the maximum distance between the endometrial-myometrial interface of the anterior and posterior walls of the uterus in the median longitudinal plane (Zhao *et al*., 2012). Sonography facilitates precise measurements of structural thickness in various anatomical contexts (Mohammadi & Mohammadi, 2023). Transvaginal sonography was employed by obstetricians for accurate measurements. A high-frequency transvaginal ultrasound probe operating at a frequency of 8 MHz was utilized. To maintain patient safety and aseptic conditions, the probe was sterilized and covered with a disposable sheath. The measurement was performed 11-12 hours prior to hCG injection using Doppler Ultrasound (E-CUBE 9, Alpinion Medical Systems Co., Ltd., Seoul, Republic of Korea). Before the procedure, patients should have a full bladder. The patient was positioned on an examination table, with the pelvic area exposed. Careful insertion of the transabdominal probe was performed by the sonographer. The probe was connected to a high-resolution ultrasound machine. During the examination, the identification of the thickest segment of the endometrium was carried out, and measurements were taken at multiple points to ensure accuracy.

### Data collection

Body mass index (BMI; kg/m^2^) was calculated as the weight (kg) divided by the square of height (m^2^). Systolic blood pressure (SBP) and diastolic blood pressure (DBP) were measured using a cuff mercury sphygmomanometer following 10 minutes of rest. Blood hypertension was identified in patients who exhibited SBP values greater than or equal to 130 mmHg and/or DBP of 85 mmHg or higher. Additionally, patients who had a history of hypertension and were receiving anti-hypertensive drug treatment were also classified as having blood hypertension. Gestational hypertension was identified when the blood pressure exceeded 140/90 mm Hg in a woman who had normal blood pressure prior to 20 weeks of gestation and lacked proteinuria, (Hasan *et al*., 2020). Preeclampsia was diagnosed when a patient with gestational hypertension also presented with proteinuria (Xiong *et al*., 2002). The investigation for diabetes involved assessing fasting blood sugar (FBS), considering a value of 95 mg/dL or higher, or the use of medication for elevated glucose levels. Gestational diabetes was diagnosed if two or more of the following criteria were met: FBS of 95 mg/dL or higher, a one-hour oral glucose tolerance test result of 180 mg/dL or higher, a two-hour result of 155 mg/dL or higher, or a three-hour result of 140 mg/dL or higher (Shokri *et al*., 2022).

### Statistical analyses

Continuous variables are presented as mean (SD), while categorical data are described as absolute numbers (%). To compare the means of continuous variables, independent t-tests were utilized. Differences in categorical variables among the study subgroups were examined using χ2 tests. Furthermore, a multivariable linear model was developed to predict neonates’ birth weight (BW) incorporating maternal characteristics and neonates’ sex as predictor variables. The level of significance was set at two-tailed α=0.05. All statistical analyses were carried out using R version 4.0.2 for Windows (https://www.r-project.org/).

## RESULTS

Of 219 included patients, 23 women were still pregnant, 12 had thyroid disease, 11 developed gestational diabetes, 11 were diagnosed as having gestational hypertension, 31 conceived twins or multiple pregnancies, 22 were users of donated oocytes, and 9 had incomplete records. The analytic sample included 100 patients with a mean (SD) age of 32.8 (6.2) years and a minimum and maximum age of 19 and 46 years. Table 1 shows the characteristics of the study sample.

**Table 1:** Maternal characteristics of the study sample (n=100).

Characteristic	Mean (SD) or Count (%)
Age (year)	32.8 (6.2)
Baseline BMI (kg/m2)	25.5 (2.5)
SBP (mmHg)	117.4 (7.2)
DBP (mmHg)	82.3 (6.6)
FBS (mg/dL)	89.0 (8.7)
Initial EMT (mm)	4.2 (1.0)
EMT During Embryo Transfer (mm)	9.1 (1.2)
EMT ≥ 8 mm (%)	85 (85.0)
EMT ≥ 9 mm (%)	58 (58.0)
Sex of the Neonate (male) (%)	37 (50.0)
Gravidity 0 (%)	66 (66.0)

* significant at *p*<0.05.

BMI, Body Mass Index; SBP, Systolic Blood Pressure; DBP, Diastolic Blood Pressure, FBS, Fasting Blood Sugar; EMT, Endometrial Thickness.

**Figure 1:**
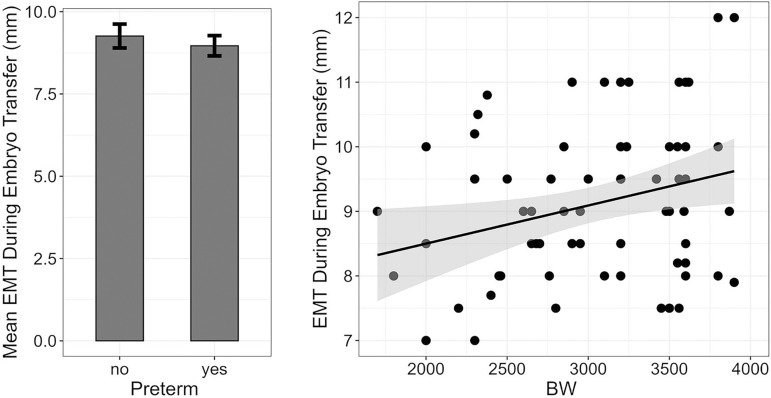
(Left) The relationship between mean EMT and preterm labor. Error bars represent 95%CI for mean estimation. (Right) The association between EMT and BW. The black line represents linear regression and the shaded gray area displays the confidence interval around the regression line. There is a positive linear relationship between EMT and BW. The narrow confidence interval shows that the regression line fits the data well. EMT, Endometrial Thickness; BW, Birth Weight.

The mean EMTs were 9.2 (1.2) and 9.0 (1.1) mm in non-preterm and preterm neonates, respectively. Univariate analysis showed that there was no statistically significant relationship between the EMT and preterm labor [*t*(93.4)=1.248, *p*=0.215, Cohen’s *d*(95%CI)=0.26 (-0.15, 0.66). Similarly, we did not find a significant association between the EMT and stillbirth or IUFD [9.1 (1.2) *vs*. 9.1 (1.1) mm in alive and dead neonates, respectively; *t*(50.5)=0.151, *p*=0.880, *d*=0.04 (-0.51, 0.59)]. However, there was a significant correlation between the EMT and the BW, Pearson’s *r* (95%CI)=0.3 (0.1, 0.5), *t*(72)=2.404, *p*=0.018. Figure 1 illustrates the relationships between EMT and preterm labor or BW.

To investigate the effects of EMT on BW we developed a multivariable linear model for predicting BW adjusted for age, baseline BMI, FBS, gravidity, SBP, and neonate’s sex. The stepwise model selection was performed using Akaike Information Criterion. Table 2 presents the result of the linear regression, *F*(4, 69)=3.850, *p*=0.007, adjusted R squared = 0.135. Overall, the model implied that EMT was a significant predictor of BW. Although maternal FBS and age were not significant predictors of neonatal BW, they approached significance with a *p* value of 0.068 and 0.092. We investigated the criteria for EMT of 8 and 9 mm concerning pregnancy outcomes (Table 3). Our analyses showed that EMT ≥8 was a significant predictor of AGA, while no other comparisons reached statistical significance. As a conservative approach, our results indicated that an EMT of ≥9 was not associated with adverse pregnancy outcomes. However, an EMT of ≥8 was sufficient to ensure a safe pregnancy.

**Table 2:** Linear model specification for predicting BW using stepwise predictor selection.

Predictor	B (SE)	t	p
Intercept	242.4 (1088.1)	0.223	0.824
EMT	124.6 (51.6)	2.416	0.018^*^
FBS	13.0 (7.0)	1.854	0.068
Age	-17.2 (10.0)	-1.710	0.092
BMI	42.3 (27.8)	1.525	0.132

* significant at *p*<0.001

EMT, Endometrial Thickness; FBS, Fasting Blood Sugar; BMI, Body Mass Index.

**Table 3:** Perinatal outcomes.

Outcome	Total	EMT ≥8 (n=15)	EMT <8 (n=85)	*p*	EMT ≥9 (n=42)	EMT <9 (n=58)	*p*
BW (g)	3,040.7 (565.3)	2,873.6 (719.8)	3,069.9 (535.7)	0.405	2,919.1 (590.7)	3,133.4 (533.7)	0.112
LBW (%)	15 (20.3)	5 (45.5%)	10 (15.9%)	0.065	9 (28.1%)	6 (14.3%)	0.240
SGA (%)	10 (13.9)	4 (36.4%)	6 (9.8%)	0.062	6 (18.8%)	4 (10.0%)	0.469
AGA (%)	56 (77.8)	5 (45.5%)	51 (83.6%)	0.016^*^	22 (68.8%)	34 (85.0%)	0.173
LGA (%)	6 (8.3)	2 (18.2%)	4 (6.6%)	0.489	4 (12.5%)	2 (5.0%)	0.475
Preterm (%)	53 (53.0)	10 (66.7%)	43 (50.6%)	0.384	23 (54.8%)	30 (51.7%)	0.922
Stillbirth or IUFD (%)	26 (26.0)	4 (26.7%)	22 (25.9%)	0.999	10 (23.8%)	16 (27.6%)	0.640

* significant at *p*<0.05

BW, Birth Weight; LBW, Low Birth Weight; SGA, Small for Gestational Age; AGA, Appropriate for Gestational Age; LGA, Large for Gestational Age; GA, Gestational Age; IUFD, Intra-Uterine Fetal Death.

## DISCUSSION

We conducted this study to investigate the effects of EMT during embryo transfer on infant BW and the rate of preterm labor. Our study included patients without any pregnancy complications, and all participating mothers had an EMT of at least 7 mm at the time of embryo transfer. Our findings indicated that there was no statistically significant association between the EMT and the occurrence of preterm labor. Similarly, we did not observe a significant association between the EMT and the incidence of stillbirth or IUFD. However, we identified a significant correlation between the EMT and BW. The multivariable linear model, adjusted for age, baseline BMI, FBS levels, gravidity, SBP, and the sex of the neonate, confirmed the significant role of EMT in predicting neonatal BW. In our study, we evaluated two criteria for EMT, specifically 8 mm and 9 mm, in relation to pregnancy outcomes. Our analyses revealed that an EMT of ≥8 mm was a significant predictor of appropriate-for-gestational-age (AGA) neonates, while none of the other comparisons reached statistical significance. Taking a conservative approach, our results indicated that an EMT of ≥9 mm is not associated with adverse pregnancy outcomes. However, an EMT of ≥8 mm was considered sufficient to ensure a safe pregnancy. Some of our findings are consistent with those reported in the existing literature.

Researchers investigated the roles of EMTs on pregnancy outcomes (Ma *et al*., 2017; Martel *et al*., 2021; Shaodi *et al*., 2020; Zhang *et al*., 2019; Zhang *et al.*, 2023). In one study, after adjusting for maternal age and BMI, a multivariate logistic model identified a significant association between EMT and live birth rate with a lower live birth in women with EMT ≤8 mm (Ma *et al*., 2017). In another study conducted by Shaodi *et al.* (2020), the impact of EMT on the embryo transfer day on pregnancy outcomes was assessed. A multivariable model was adjusted for the age, duration of infertility, BMI, infertility type, and number and type of embryos transferred. They found a significant correlation between the EMT ≥8.7 mm and the live birth rate and concluded that the live birth rate would be reduced with a very thin endometrium. (Shaodi *et al*., 2020). In a recent retrospective cohort study, Martel *et al.* (2021) investigated the effects of EMT on BW and obstetric complication rate after hormone-replaced frozen-ET. The median endometrial thickness was 8.60 mm. The sample was dichotomized into two groups of EMT <7 and ≥7 mm. Neonates born from EMT <7 mm were born earlier and with lower BW (2,749.9 *vs*. 3,345.2 g). Nevertheless, the EMT was not significantly associated with obstetric complications, even when adjusted for age and medical history. They showed that EMT was not associated with the incidence of preterm birth (*p*=0.67) and that the rates of preterm birth were not statistically different in women with a thin *versus* thick endometrium (28.6% *vs*. 8.5%, *p*=0.12). Moreover, there was no significant difference in rates of SGA infants between mothers with an EMT of <7 and those with an ETM of ≥7 mm (20.0% *vs*. 3.0%, *p*=0.15). Martel *et al*. (2021) reported that EMT was significantly correlated with birth weight (*p*<0.03). In a study conducted by Zhang *et al*. (2023) on frozen-ET, the mean birth weight was 85.1 g higher in the EMT ≥ 12 mm group and 25.9 g higher in the 8-12 mm EMT group than in the EMT <8 mm group. They concluded that the BW is lower for newborns delivered by patients with a thinner endometrium. A significant difference in BW has been reported in another study comparing EMT <8 and EMT ≥10 mm (Zhang *et al*., 2019).

Our study particularly replicated some of Martel’s results (Martel *et al*., 2021). While our model was adjusted for the known confounders of BW, we lowered the likelihood of bias at the level of the study design by restricting our sample to mothers without health unfavorable conditions. Moreover, we included patients with ≥7 mm EMT for embryo transfer. These allowed us to select a more homogenous sample and lower the effects of factors interacting with normal BW. In our multivariable model, we incorporated EMT as a continuous variable instead of a dichotomous feature. This prevented the loss of information by categorizing EMTs. However, our univariate analysis did not reveal an important difference in the outcomes of pregnancy using EMT cutoffs of 8 and 9 mm. Overall, the mechanism underlying the relationship between EMT and neonatal BW is multifactorial. However, it is believed that a thin endometrium may lead to higher oxygen tension and increases exposure to reactive oxygen species that can be unhealthy for the fetus (Casper, 2011). Thin endometria lack a functional layer which means that the fetus is exposed to the large spiral arteries and increased oxygen tension, often resulting in a lack of oxygen supply (Casper, 2011). Deficiencies in deep placentation and remodeling of arteries as a result of a thin endometrium can adversely affect blood flow to the placenta and uteroplacental circulation, ultimately affecting the developing fetus (Zhang *et al*., 2019). Meanwhile, additional research is still needed to investigate how the thickness of the endometrium is associated with specific obstetric complications and to select the optimal EMT cutoff for clinical decision-making.

### Limitations

The study presented in this manuscript has some limitations that should be considered when interpreting the results. First, this study was a retrospective cohort, and the sample was limited to patients treated at a single center. Consequently, the generalizability of the findings to a broader population may be limited. Furthermore, we assessed short-term outcomes and did not investigate long-term effects, such as the potential impact of EMT on child development or long-term health outcomes. Conducting a long-term study would provide a valuable complement to the assessment of the safety of existing EMT criteria. Moreover, while we attempted to control for potential confounding factors in our statistical analysis, there may still be unmeasured confounders that could affect the results. A larger sample size is necessary when incorporating a substantial number of covariates. In our study, we specifically included mothers who did not have significant obesity, HTN, or diabetes. To establish and generalize our findings, further prospective longitudinal studies with larger sample sizes are warranted. Particularly, the recruitment of specific patient groups, such as individuals with pregnancy complications or baseline health conditions, and extending the follow-up period would contribute to a more comprehensive and generalized conclusion. These modifications would enable a deeper understanding of the relationship between endometrial thickness EMT and the various outcomes of interest.

We investigated the relationship between the EMT during embryo transfer and BW or preterm labor. All participating mothers had an EMT of at least 7 mm at the time of embryo transfer. The results indicated that an EMT of ≥7 mm did not demonstrate a significant association with preterm labor, and stillbirth or IUFD. However, our adjusted model revealed that the EMT plays a significant role in predicting newborn weight. In a conservative approach, we found that newborns resulting from embryo transfers with maternal EMT ≥9 mm did not show an increased risk for unfavorable pregnancy outcomes. However, our study revealed that EMT ≥8 mm would be safe for newborn health as well. These findings suggest that maintaining an EMT of at least 8 mm during embryo transfer is associated with favorable outcomes for newborns, while an EMT of 9 mm or more reduces the likelihood of adverse pregnancy outcomes to a greater extent.

## Highlights

- EMT plays a significant role in predicting newborn weight.
- An EMT of ≥7 mm did not demonstrate a significant association with preterm labor.
- An EMT of ≥7 mm did not increase the risk of stillbirth or IUFD.
- Conservatively, an EMT ≥9 mm did not increase the risk of pregnancy complications.
- An EMT ≥8 mm would be safe for the newborn health as well.

## Abbreviations

ART: Assisted Reproductive Technology

BMI: Body Mass Index

BW: Birth Weight

DBP: Diastolic Blood Pressures

ET: Embryo Transfer

FBS: Fasting Blood Sugar

GA: Gestational Age

hCG: human Chorionic Gonadotropin

LBW: Low Birth Weight

LGA: Large for Gestational Age

SBP: Systolic Blood Pressures

SD: Standard Deviation

SGA: Small for Gestational Age
